# Global inventory of suitable, cultivable and available cropland under different scenarios and policies

**DOI:** 10.1038/s41597-022-01632-8

**Published:** 2022-08-27

**Authors:** Julia M. Schneider, Florian Zabel, Wolfram Mauser

**Affiliations:** Department of Geography, Ludwig-Maximilians-Universität München, Munich, Germany

**Keywords:** Environmental impact, Climate-change impacts, Sustainability, Environmental impact

## Abstract

Where land-use change and particularly the expansion of cropland could potentially take place in the future is a central research question to investigate emerging trade-offs between food security, climate protection and biodiversity conservation. We provide consistent global datasets of land potentially suitable, cultivable and available for agricultural use for historic and future time periods from 1980 until 2100 under RCP2.6 and RCP8.5, available at 30 arc-seconds spatial resolution and aggregated at country level. Based on the agricultural suitability of land for 23 globally important food, feed, fiber and bioenergy crops, and high resolution land cover data, our dataset indicates where cultivation is possible and how much land could potentially be used as cropland when biophysical constraints and different assumptions on land-use regulations are taken into account. By serving as an input for land-use models, the produced data could improve the comparability of the models and their output, and thereby contribute to a better understanding of potential land-use trade-offs.

## Introduction

Looking at the challenges of the 21^st^ century that are addressed in the United Nations’ Sustainable Development Goals (SDGs)^1^, land plays a major role in various SDGs, such as in meeting the increasing demand for food and bioenergy, the conservation of ecosystems and biodiversity or taking action against climate change. However, competing needs of land and emerging trade-offs between the different usages increase the pressure on land, which is particularly apparent in the context of agricultural land-use: While expansion and shifting agriculture were identified as main drivers for biodiversity loss^2–5^ and deforestation^6–8^, which is moreover accompanied with carbon emissions^9–12^, cropland extent is still projected to increase globally by up to 7% until 2050^13^.

Thus, investigating land-use change and particularly the spatial dynamics of potential future cropland expansion is an important current research task, which can be addressed with land-use models and Integrated Assessment Models^14–19^. Thereby, information on the extent and the spatial location of potentially cultivable land resources that could be transformed into cropland is an essential input information^20^. Depending on the model, it constrains for example the spatial extent of cropland expansion^21^ or impacts the costs of land conversion and land prices^20,22^. However, the spatial location and extent of land assumed to be potentially cultivable differs between models, mainly due to different assumptions on which current land-use/-cover is considered as being potentially available for cropland use^20^. Yet, a study from Eitelberg *et al*.^20^ shows that also variations of up to 84% exist between cultivable land datasets with comparable assumptions on land availability, resulting from different underlying data and also from differences in assumed biophysical constraints for cropland use. Depending on the sensitivity of the land-use model, those differences can have large implications on the simulated results and thus complicate the comparison of model outputs and simulated land-use change projections.

Reference data on potentially cultivable land could contribute to increasing the comparability and consistency between land-use change simulations^23,24^. However, published datasets are rare, and due to their often very specific assumptions difficult to apply within different models and studies. They refer for example only to specific crops when evaluating the biophysical suitability of land for agriculture^25–27^, include various social, administrative and economic constraints^28^, focus only on a specific time period or region^28^, or are provided in a rather coarse spatial resolution^20^. To be applicable in as many land-use models and scenarios as possible, a dataset of potentially cultivable land should (1) include a broad spectrum of agricultural crops when evaluating the suitability of land for agriculture. It is (2) ideally provided with and without universally applicable assumptions on institutional restrictions constraining the availability of land for agricultural use, and is (3) available in a high spatial resolution on a global scale and for different time periods, considering also climate change.

**Fig. 1:**
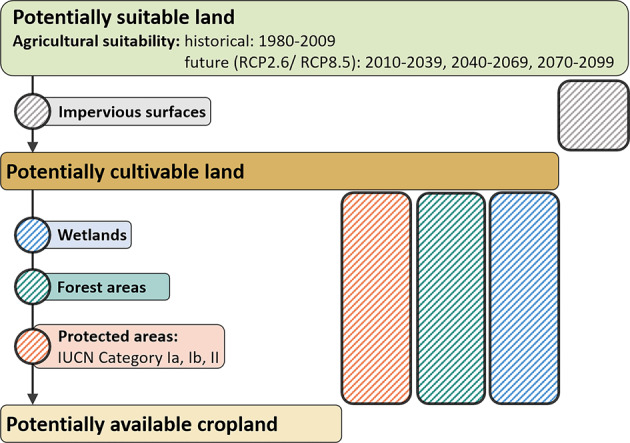
Overview of the analysis framework. The agricultural suitability refers to land that is suitable for crop cultivation under the environmental conditions of each time period and climate scenario. The potentially cultivable land excludes impervious areas that are agriculturally suitable, such as settlements or roads. The potentially available cropland additionally accounts for a subset of institutional restrictions, regulations and (potential) nature protection policies on the agricultural use of land. Thereby, we assume strictly protected areas, forests and wetlands to be not available for cropland use, and thus exclude those areas in the potentially available cropland.

Here, we provide two spatially explicit global datasets: An updated version of the agricultural suitability^29^, and a new dataset on potentially cultivable and available land for agricultural use^30^ for past and future time periods from 1980 until 2100^29,30^. While biophysical and climatic constraints determine the potential suitability for agriculture, the potentially cultivable land is defined by its agricultural suitability and the (technical) feasibility of crop cultivation. The potentially available cropland additionally takes selected existing and potential nature protection policies into account (Fig. 1).

The assessment of potentially cultivable land is based on the historic and future agricultural suitability for 23 food, feed, fiber, and first- and second-generation bioenergy crops^31,32^ considered as globally important with respect to their cultivation area and production volumes (Table 1). By using a fuzzy logic land suitability model^32^, the approach accounts for crop-specific characteristics and requirements during the growing period with regard to climate, soil and topography, and considers the effects of climate change on the agricultural suitability for two representative concentration pathways, RCP2.6 and RCP8.5^33^. The impact of irrigation on the suitability is taken into account by referring to current irrigation patterns^34^, which we assume constant also for future time periods. This results in a dataset on land potentially suitable for agricultural use (for details see Methods). We use high resolution data on man-made impervious areas^35^ to exclude settlements and infrastructure, which we assume to be technically difficult or unlikely to be cultivated or converted into cropland. The resulting dataset displays the potentially cultivable land. Thereupon, the potentially available cropland dataset is created by considering the most strictly protected areas^36^, designated with the International Union for Conservation of Nature (IUCN) Category Ia, Ib and II, as not available for cropland use. Moreover, we exclude forests^37^ and agriculturally suitable, but not yet cultivated wetlands^38^ from the potentially available land due to their importance for carbon sequestration and biodiversity conservation. Thus, the dataset accounts for a subset of selected (institutional) restrictions, regulations and (potential future) policies on cropland expansion and nature protection, thereby reflecting key aims of the Sustainable Development goals^1^ and recent efforts to stop deforestation^39^, protect the climate^40^ and preserve biodiversity^41^ (Fig. 1). Yet, it is important to note that the term ‘potentially available cropland’ does not imply that the identified land can unrestrictedly be used for crop cultivation without potentially arising conflicts and trade-offs with other land-uses (see the Discussion section for details).

**Table 1:** Overview of the considered crops within the agricultural suitability.

Food, feed, fiber and first-generation bioenergy crops			Second-generation bioenergy crops
Barley	Potato	Sugarbeet	Jatropha
Cassava	Rapeseed	Sugarcane	Miscanthus
Groundnut	Rice	Sunflower	Switchgrass
Maize	Rye	Summer wheat	Reed canary grass
Millet	Sorghum	Winter wheat	Eucalyptus
Oilpalm	Soy		Willow

Included are staple crops of global importance, such as maize, wheat and rice, that provide more than half of global calorie intake, but also more regionally important food crops, such as millet or cassava. Furthermore, we include the main first- and second-generation bioenergy crops to capture the trends in political support of biofuels and the emerging bioeconomy.

All resulting global datasets on potentially suitable, cultivable and available land are available for historic (1980–2009) and three different future time periods (2010–2039, 2040–2069, 2070–2099) under RCP2.6 and RCP8.5 at 30 arc-seconds (approximately 1 km at the equator) spatial resolution, and thus allow for historic and future land-use change analysis under different climate change scenarios. By including first- and second-generation bioenergy crops into our suitability assessment, also land-use change and cropland expansion in the context of an emerging bioeconomy can be investigated. Moreover, the datasets are available for rainfed and irrigated conditions separately to enable the investigation of land-use change under changing irrigation patterns. Since the information of potentially cultivable land resources could also be of interest for models that use aggregated data, such as many economic models^42^, all datasets are available also in aggregated form at country level. The provided data could thus contribute to increase also the consistency within interdisciplinary research and integrated model coupling approaches investigating land-use change and arising trade-offs.

## Results

### Historic potentially cultivable land and potentially available cropland

**Fig. 2:**
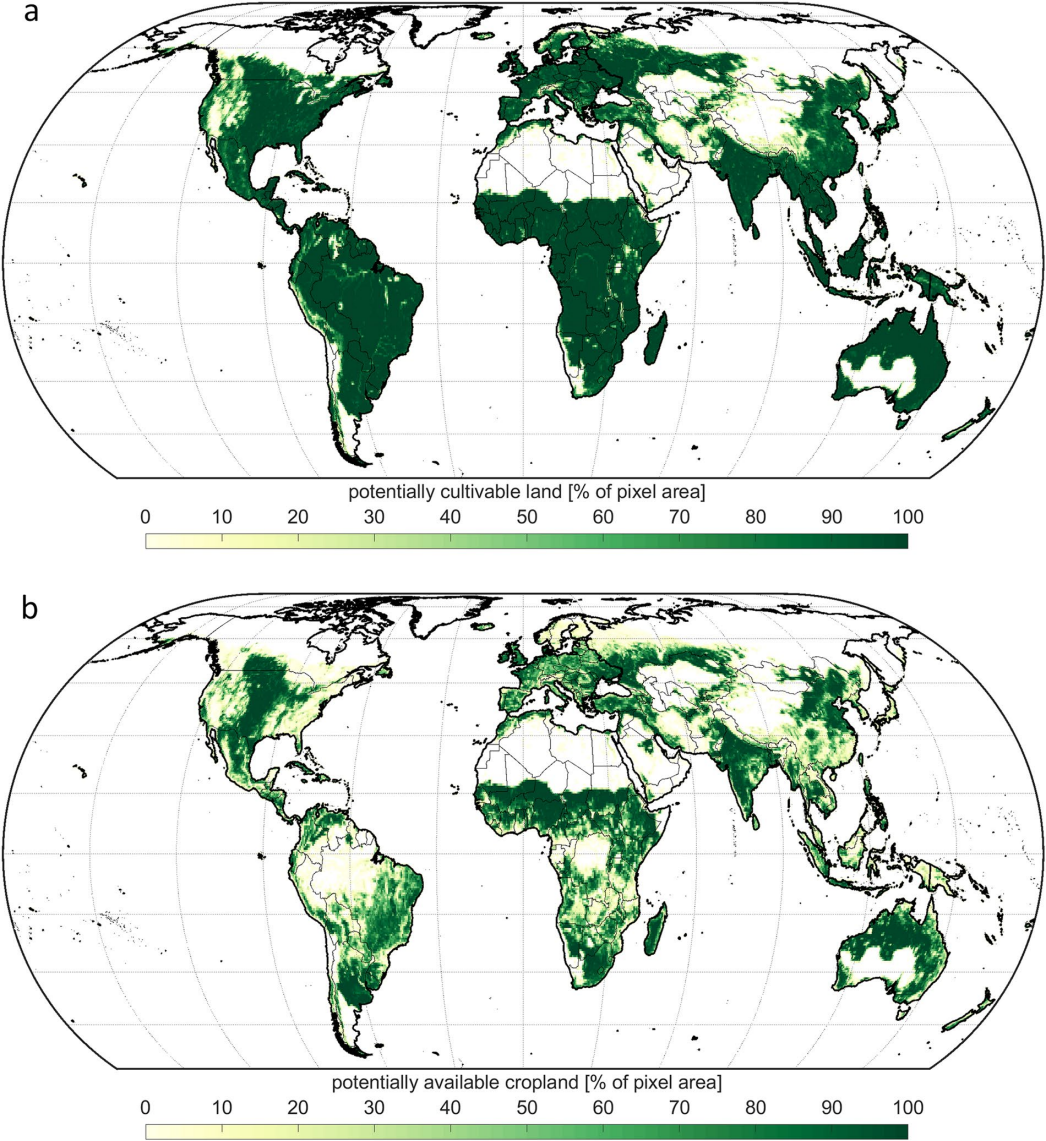
Global potentially cultivable land (**a**) and potentially available cropland (**b**) for historic time period. The maps display (**a**) the share of potentially cultivable land on the total area of each 1 km pixel [%], taking only biophysical restrictions for cultivation into account and (**b**) the share of potentially available cropland on the total pixel area [%] additionally considering restricted agricultural use of forests^37^, wetlands^38^ and protected areas^36^.The country borders are displayed according to the global administrative areas of GADM version 3.6 (https://gadm.org/).

For the historic time period (1980–2009), around 78.1 million km^2^ are potentially suitable for agricultural use under current irrigation patterns. Of this potentially suitable land, around 390,000 km^2^ (0.5%) are impervious surfaces such as human settlements or infrastructure, and thus considered as being not cultivable. Accordingly, globally around 77.7 million km^2^ are potentially cultivable in terms of biophysical characteristics regarding soil, climate, topography and the current surface cover (Fig. 2a). Of this area, 3% is designated as strictly protected area, mainly covered with forests (64%), while additionally 36% of the potentially cultivable land is covered with forests and 1% with wetlands not classified as strictly protected, resulting in a potentially available cropland of around 46.3 million km^2^ (Fig. 2b).

**Fig. 3:**
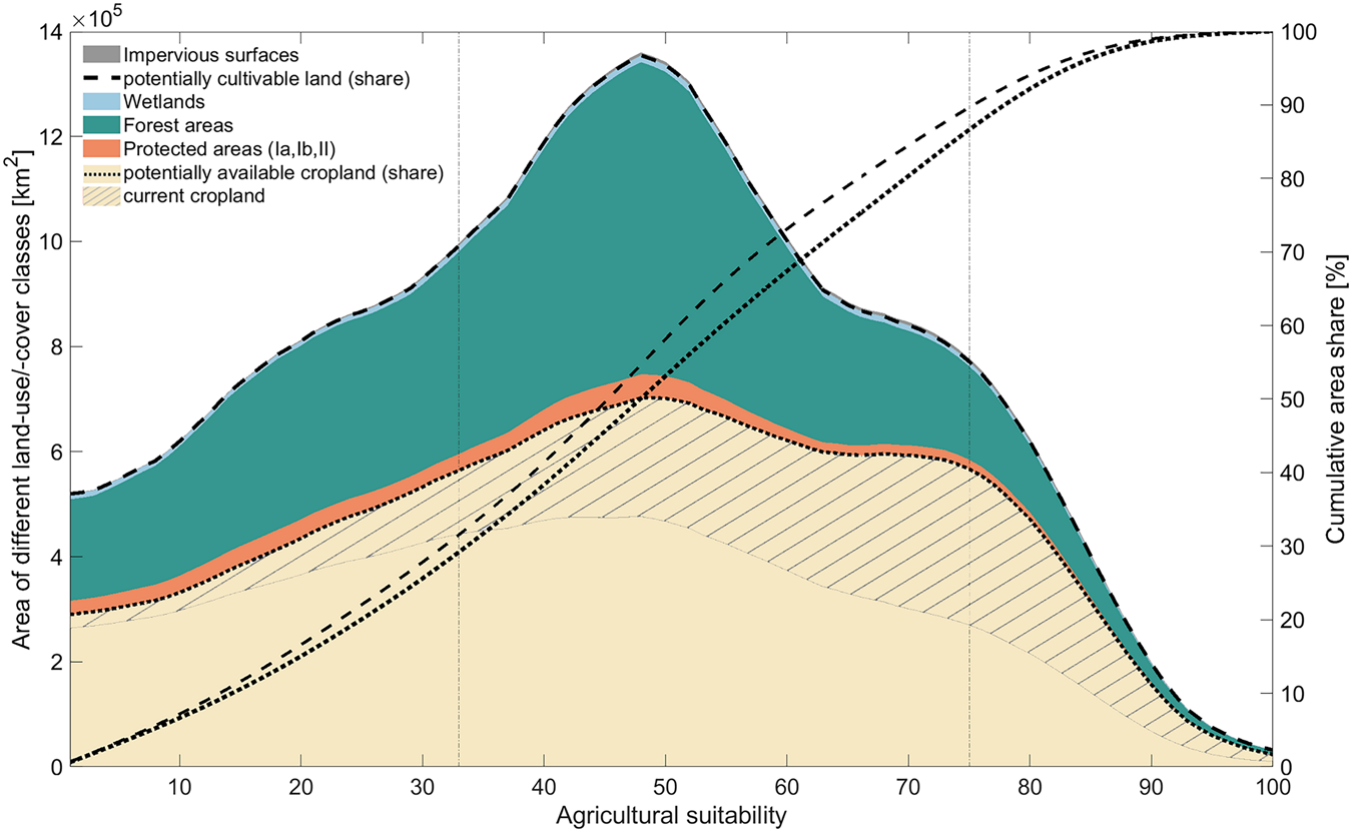
Suitability and land-use/-cover of the potentially cultivable land and potentially available cropland. The area graph shows the potentially suitable area [km^2^] and the different land-use/-cover classes (displayed as moving average) considered to distinguish between the potentially cultivable land and the potentially available cropland dependent on the agricultural suitability. Hatched areas indicate the parts of the potentially available cropland already under cultivation^37^. The potentially cultivable land and potentially available cropland area [km^2^] for each suitability value is indicated with the dashed and dotted line inserted in the area graph. The two additional line graphs display the cumulative distribution function of the potentially cultivable land (dashed line) and potentially available cropland (dotted line), respectively. They thus visualize the cumulative area share in the total area potentially cultivable and available [%] across the agricultural suitability.

Around 10% of the potentially cultivable land is highly suitable for agriculture (suitability values: 75–100), while the major part of it (58%) is moderately suitable (suitability values: 33–74) and around one third (32%) is marginally suitable (suitability values: 1–32) for agriculture (Fig. 3). By excluding land currently covered with forest, wetlands and protected areas to assess the potentially available cropland, mainly marginally (35%) and moderately (60%) suitable areas are excluded. Yet, around 1/3 (33%) of the 46.3 million km^2^ of potentially available cropland is currently already used as cropland^37^. Since current cropland is mainly located in highly and moderately suitable areas, 35% of the potentially available cropland not yet under cultivation is marginally suitable for agriculture (Fig. 3).

**Fig. 4:**
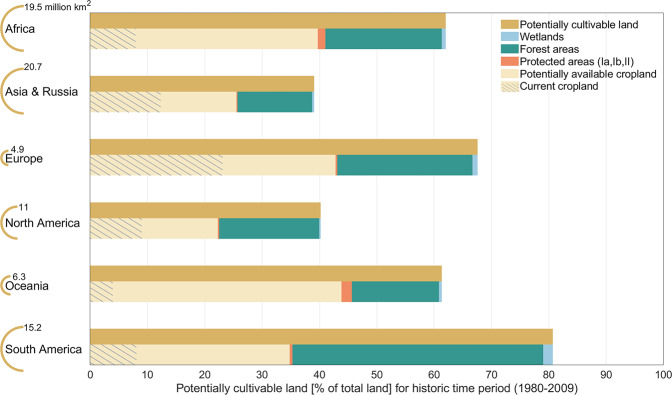
Regional potentially cultivable land [% of total land] for the historic time period. The bars display the share of potentially cultivable land in total land area [%], the shares of protected areas^36^, forested areas^37^ and wetlands^38^ and the resulting potentially available cropland. The hatched parts of the bars indicate the potentially available cropland currently already cultivated^37^. The absolute area of potentially cultivable land is displayed in million km^2^ for each world region within the legend of the y-axis. The region mapping is displayed in the Methods section (Fig. 7).

Regarding the regional potentially cultivable land resources, South America shows with around 81% globally the largest share of cultivable land in total land area. It is followed by Europe (68%) and Africa (62%), while in North America and Asia & Russia less than half of the total land area is potentially cultivable (Fig. 4). Yet, especially in South America, Europe and North America, large shares of the total land area are covered with forests: For example, in South America, 46% of the land area is covered with forests, of which 94% is potentially suitable for agriculture. Accordingly, when excluding forests, wetlands and protected areas to assess the potentially available cropland, the potentially cultivable land is more than halved to 35% of total land area being potentially available for cropland. In Europe, North America and Asia & Russia, the regions with the globally second, third and fourth largest share of forested land in total land area, 85%, 62% and 48% of the forest areas are potentially suitable for agriculture. Excluding forest areas together with wetlands and protected areas thus reduces the potentially cultivable land by around 37%, 45% and 35%, respectively, to a share of potentially available cropland in total land area of 43% in Europe, 22% in North America and 26% in Asia & Russia. Due to the generally small area of forests and protected areas in Oceania, the share of potentially cultivable land (61%) is only marginally reduced and 44% of the total land area is potentially available for cropland use (Fig. 4).

The potentially cultivable land as well as the potentially available cropland entail land currently already under agricultural use. Particularly in Europe, Asia & Russia and North America, large parts of the potentially available cropland are already used as cropland: 54% in Europe, 48% in Asia & Russia and 40% in North America (Fig. 4). In South America, Africa or Oceania on the other hand, only about 23%, 20% and 9% of potentially available cropland is currently in use. Accordingly, especially in Africa and South America, but regarding absolute land resources also in Asia & Russia, large areas of approximately 10 million km^2^ (Africa), 5 million km^2^ (South America) and 7 million km^2^ (Asia & Russia) would potentially remain for a transformation into cropland. Yet, regarding the suitability of the potentially available cropland that is not yet agriculturally used, highly suitable remaining land resources are mainly located in Africa (1.28 million km^2^), Asia & Russia (768,000 km^2^) and North America (454,000 km^2^), where around 11% to 13% of the potentially available cropland not yet under cultivation is highly suitable for agriculture. In South America and Oceania on the other hand, large parts of the potentially available cropland not yet under cultivation, 40% and 39%, respectively, are marginally suitable.

### Change of potentially cultivable land over time until future time period 2070–2099

**Fig. 5:**
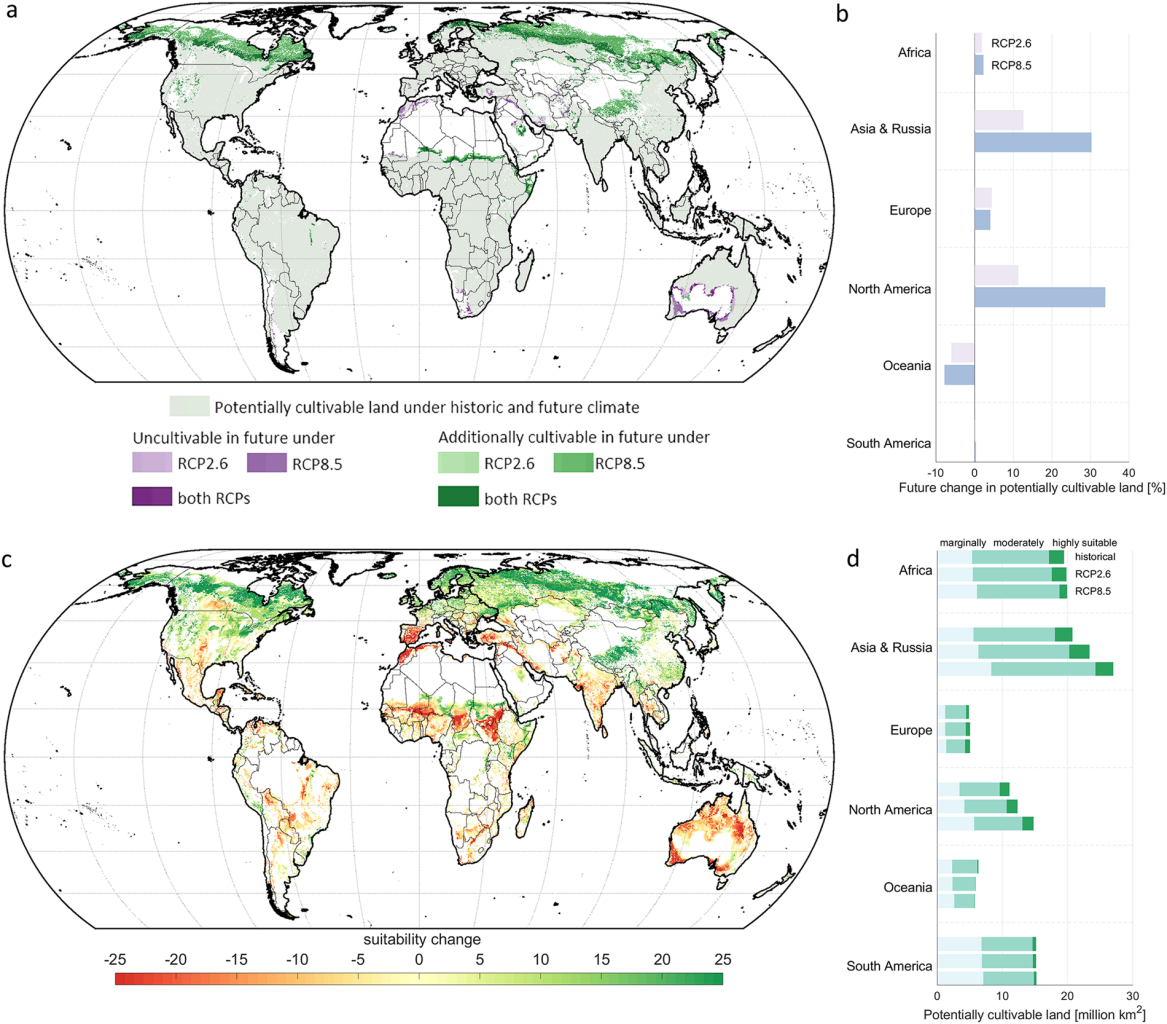
Future changes in potentially cultivable land extent and its suitability until the end of the century (2070–2099) compared to the historic time period (1980–2009). Map (**a**) indicates regions that become potentially cultivable in the future under RCP2.6, RCP8.5 or under both scenarios, as well as regions that lose their suitability for agriculture under future climate and thus are not cultivable anymore under one or both climate change scenarios. Graph (**b**) displays the percentage change [%] of potentially cultivable land between the historic and the future time period (2070–2099) under both RCPs within each world region. Map (**c**) shows the underlying change in agricultural suitability from historic time period until 2100 exemplarily for potentially cultivable land under RCP8.5. Graph (d) displays the absolute potentially cultivable land area [million km^2^] for historic and both future time periods, subdivided into marginally suitable (light green), moderately suitable (green) and highly suitable land (dark green).

Until 2100, the potentially cultivable area globally increases compared to the historic time period by 5% and 13% under RCP2.6 and RCP8.5, respectively, resulting in a potentially cultivable land area between 82 million and 88 million km^2^. The largest increases in cultivable land compared to the historic time period can be found in North America and Asia & Russia with +11% and +12%, under RCP2.6, and with +34% and +30%, respectively, under RCP8.5 (Fig. 5a,b). This increase mainly results from a northwards shift of the agricultural frontier due to global warming. Thus, large areas in the northern hemisphere become at least marginally suitable for agriculture. Large parts of these areas are currently covered with forests, leading to an increase of forest areas being assessed as potentially cultivable from around 29.8 million km^2^ in historic time period by +7% to 31.7 million km^2^ under RCP2.6, and by +18% to 35 million km^2^ under RCP8.5 until 2100. Mainly in North America and Asia & Russia, forest areas being potentially cultivable increase by +16% in both regions under RCP2.6 and by +49% in Asia & Russia and by +40% in North America under RCP8.5. Moreover, also areas currently covered with wetlands become suitable for cultivation until 2100. Compared to historic time period, the area of current wetlands potentially cultivable globally increases by 25% until 2100 under RCP2.6 and by almost 70% under RCP8.5. Similar to the observed changes in forest suitability, the area of potentially cultivable wetlands increases mainly in the northern latitudes of Asia & Russia (+272%) and North America (+114%). Overall, the additional agriculturally suitable areas in the north outweigh the areas that become unsuitable in the future, e.g. by becoming too hot or too dry for agriculture. The potentially cultivable land area remains widely constant in Africa and South America under both climate change scenarios, while it decreases until 2100 between −6% and −8% in Oceania under RCP2.6 and RCP8.5 (Fig. 5a,b).

Beside changes in the extent of the potentially cultivable land, also its agricultural suitability changes due to changing future climate conditions (Fig. 5c,d). Under RCP8.5, we find an increase in potentially cultivable land that is marginally suitable (+26%), while highly suitable land decreases by −11%. This decrease can particularly be observed in Africa, where the potentially cultivable land highly suitable for agriculture halves (−51%), whereas marginally suitable land increases by +14%. Accordingly, the share of highly suitable land on the potentially cultivable land in Africa decreases from 12% in 1980–2009 to 6% in 2070–2099 under RCP8.5. In Europe on the other hand, the contrary is the case: Even though the total potentially cultivable land increases by only +4% compared to historic time period, the share of highly suitable land increases from 9% to 16%, leading to an increase in highly suitable land area of +82% especially in northern Europe. Yet, also marginally suitable land area increases by +16%, for example in the Mediterranean, so that in total the share of moderately suitable land on potentially cultivable land in Europe decreases by 9 percentage points (pp) from 66% (1980–2009) to 57% (2079–2099). In North America, where potentially cultivable land increases by +34%, mostly marginally suitable land in Canada becomes cultivable, while the agricultural suitability of large areas decrease in the south of the United States of America and Mexico. Accordingly, the share of marginally suitable land in potentially cultivable land area rises by 7pp to 38%, while the share of moderately and highly suitable land decreases by 5pp and 2pp to 50% and 12% of potentially cultivable land. Under RCP2.6, on the other hand, where the potentially cultivable land globally increases by +5%, the shares of marginally, moderately and highly suitable land remain nearly constant on a global scale and vary only slightly on regional scale.

Our results thus show that especially for investigating future land-use change, the impact of different climate change scenarios on agricultural suitability needs to be taken into account to include land that will become suitable for agriculture in the future. Moreover, we see that due to the fact that particularly forest areas in the northern hemisphere become potentially cultivable under future climate, the assumption on forests being potentially available for cropland use or not is becoming more relevant, as its effect on the total extent of potentially available cropland increases.

## Discussion

An important question causing large differences in the estimations of potentially cultivable land is the question *‘how suitable is suitable enough?’ *^20^. By determining at each pixel the maximum suitability across all crops included in our agricultural suitability, our potentially cultivable land includes all areas that are agriculturally suitable for at least one of the 23 considered crops (Table 1). Thus, we consider also marginally suitable land for a specific crop as potentially cultivable. Since areas with a low suitability for agriculture might not be transformed into cropland, as the attainable crop yields could be too low or unstable, the attained potentially cultivable land and available cropland provide rather an upper benchmark estimate. However, within regions that are generally rather marginally suitable for agriculture, also land with a rather low suitability can be considered as suitable and thus be of interest for agricultural use. Further, a potential conversion of land into cropland is, beyond its agricultural suitability, mostly influenced by the potentially attainable yields (of specific crops) on the cultivable land. Those yields do not solely depend on the agricultural suitability in terms of soil properties, climate and topography, but might to a large extent also be influenced by soil-, crop- and farm-management measures, which are only partly considered in this suitability approach. Additionally, also the political and socio-economic framework drives land-use dynamics^43–45^: For example, depending on the scarcity of land and achievable crop prices, it might even be profitable to transform marginally suitable land into cropland, for example for the cultivation of bioenergy crops^46^. Therefore, we consider it useful to be least restrictive in our assumptions on the agricultural suitability of potentially cultivable land and thus have not applied a suitability threshold (see Methods). Model- or study-specific further biophysical limitations for cultivation can either be applied directly to our spatially explicit datasets, be implemented with a suitability threshold, or could also result from a land-use modelling framework, e.g. through simulated crop yields or economic factors such as demand or prices.

By maintaining the current irrigation patterns^34^, we avoid assuming an expansion of irrigation into areas where water might not be (sustainably) available or an implementation of irrigation infrastructure not feasible, for example due to a lack of technology, knowledge or required investments^47^. Nonetheless, as an expansion of irrigation infrastructure would substantially change global patterns of potentially cultivable land, a corresponding dataset of available cropland under irrigation provides an interesting input into land-use change models to investigate the effects and land-use dynamics of an expansion of irrigation. Therefore, we provide each dataset of potentially cultivable land and available cropland also under rainfed and irrigated conditions separately. This allows users to apply own assumptions on the expansion of irrigated areas.

Another central question is the definition of ‘*availability for cropland use’*. To provide a basis dataset that leaves aside institutional, socio-economic or policy-driven assumptions on the issue of accessibility and availability, we applied only biophysical restrictions to derive the potentially cultivable land. We thereby assume that man-made impervious surfaces are difficult and unlikely to be used as cropland due to, the associated uncertainty regarding their transformability to cropland, arising costs for their transformation into cropland and potentially resulting conflicts of use. For the assessment of the potentially available cropland, we assume that the most strictly protected areas, forests and not yet agriculturally used wetlands are not considered as available for cultivation. We have chosen these restrictions for several reasons: First, those land-use/-cover types play a key role in biodiversity conservation^48–51^ and climate change mitigation due to their large potential for carbon sequestration^52–55^ and thus in the context of the SDGs, while at the same time the conversion of forests and wetlands are land-use/-cover changes of major importance in the context of agricultural development^56,57^. Second, the applied restrictions reflect efforts of current policies and institutional regulations. Particularly a stop of deforestation until 2030 was recently declared as an important goal at the 26^th^ UN Climate Change Conference of the Parties^39^, while possibilities for agricultural activities in protected areas are regulated within the IUCN categorization of protected areas. Accordingly, assuming their unavailability for the conversion into cropland creates a dataset that reflects the importance of those land-use/-cover types and the (declared) ambitions to protect them. However, it needs to be noted that there are concepts for the cultivation of wetlands and forests which for example go beyond drainage-based agriculture and deforestation, such as Paludiculture^58^ or Agroforestry^59^, and thus enable an agricultural use that is less conflicting with aims of climate protection and biodiversity conservation. Finally, the study of Eitelberg *et al*.^20^ shows that in many of the reviewed studies and land-use models, forests are excluded from estimations of potentially available land^26–28^, suggesting that these restrictions might be most commonly applied across different models and studies. Nonetheless, further political, social, cultural and economic factors, such as land tenure issues or conversion costs of land to cropland^28^, as well as current uses of the land, e.g. as pastures or idle land, can additionally restrict or conflict its availability for crop cultivation. Thus, it strongly depends on the land-use model, the research question and the spatial scale of investigation whether, how and at which spatial level further restricting factors are exogenously or endogenously implemented^60^. Due to the spatial explicitness and the high spatial resolution of our generated datasets, further exogenous restrictions that might be needed for specific land-use models or research questions can be applied by overlaying and masking our datasets with corresponding spatial data. Users could for example extend the assumptions on potential nature protection policies and additionally exclude grasslands or specific high-biodiversity areas from the potentially available cropland, or use the potentially cultivable land data to apply own restrictions that for example only exclude currently already protected wetlands or forests.

**Fig. 6:**
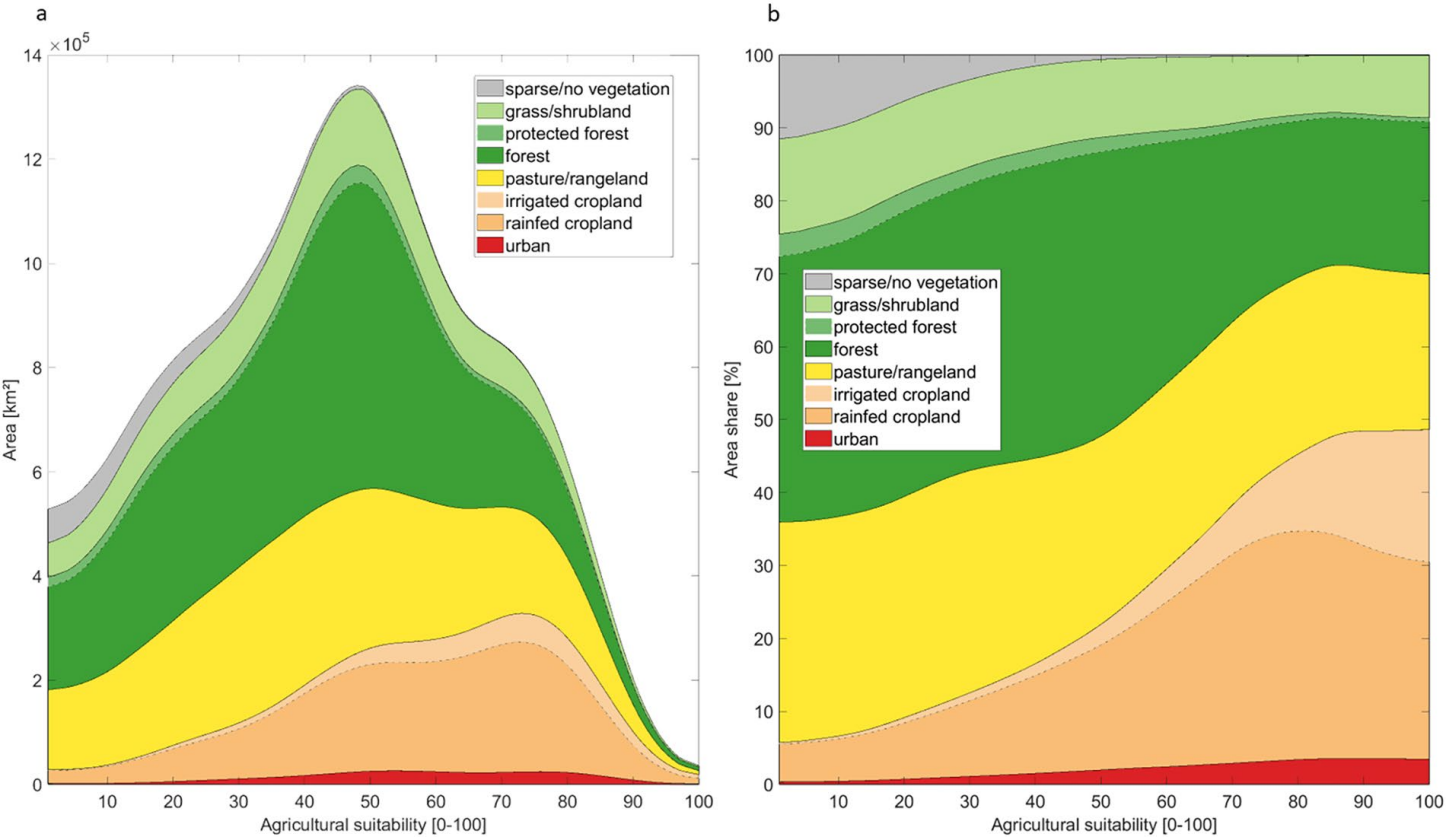
Land-use/-cover of the global agriculturally suitable areas. (**a**) Displays the absolute area [km^2^] of each land-cover class for each agricultural suitability value, while (**b**) shows the relative area share of each land-cover class. A moving average was applied in both figures. The land-use/-cover refers to HILDA+ data^37^ for the year 2010.

Overall, it is important to note that the term ‘potentially available cropland’ does not imply that the identified areas are unrestrictedly available for agricultural use without any trade-offs or potential land-use conflicts. Large parts of the identified land suitable for crop production are recently not cultivated, but covered for example with grassland or shrubland, or used as pasture (for details see Fig. 6 in the Methods section). Changing the land-cover and –use of these areas by cultivating them can have various negative implications, for example destroy valuable ecosystems and thereby the habitat of species, or reduce the carbon sequestration potential of these areas and lead to carbon emissions. Depending on the current land-use, a conversion into cropland can moreover impact the local population by taking away their livelihoods and thus force migration. A conversion into cropland can thus lead to social conflicts and compromise important action for example towards climate protection and biodiversity conservation. Moreover, further practical issues associated with land-use and cropland conversion, such as land tenure, social or cultural functions of the current land-use, the political framework and land-use regulations, or the availability of capital and labor to cultivate this land, are not considered in our approach. Yet, by providing information on land potentially cultivable or available and its agricultural suitability, our datasets can be used to identify possible conflicting areas where land is under a high risk of being transformed into cropland. The dataset can thus contribute to analyze where trade-offs between crop cultivation and other land-use and land functions could occur, and thereby point to regions where an implementation of land-use regulations might be of particular importance, especially in the context of projections on future cropland expansion. Besides that, it might be necessary to think about a change in the terminology to describe land potentially available for crop production towards a term that reflects also the potentially arising trade-offs and land-use conflicts that can come along with cultivating this land.

Assessing the potentially cultivable land for future time periods enables to include also land currently not yet suitable for agricultural use in assessments of land-use dynamics, and thus allows for consistently investigating future land-use dynamics and potential cropland expansion under a changing climate by providing realistic boundary conditions. Yet, it is important to note that future scenarios of urbanization and changes in man-made impervious surface as well as future changes in forest cover and wetlands, e.g. due to forest degradation, deforestation or afforestation or the drainage of wetlands, as well as the expansion or reallocation of protected areas could not be considered in this approach, but could also be applied upon the provided datasets.

We conclude that by providing information on potentially cultivable land and potentially available cropland for historic and different future time periods, spatially explicit and additionally aggregated to country level, our datasets could serve as basic input datasets for different types of models investigating land-use dynamics. This could improve the comparability in land-use change modelling^23,24^, which is crucial for a better understanding of land-use dynamics, feedbacks and potentially emerging trade-offs between crop production and other land-uses and functions.

## Methods

### Agricultural suitability

To identify the land that is potentially suitable for agricultural use, we refer to an updated version (v3.0) of the data on agricultural suitability by Zabel *et al*.^32^, in which updated data on soil, irrigated areas and climate were applied, and an increased range of crops was considered. The suitability of land for crop production was assessed with a fuzzy logic land suitability model^32^ at 30 arc-seconds spatial resolution for 23 globally important crops in terms of their cultivation area and production volume (Table 1). Among those 23 crops, the 17 food, feed, fiber and first-generation bioenergy crops currently represent around 67% of global harvested area and 73% of the global production volume according to FAOSTAT^61^, while jatropha, miscanthus, switchgrass, reed canary grass, eucalyptus and willow represent six important second-generation bioenergy crops.

The land suitability model accounts for crop-specific characteristics and requirements during the growing period with regard to climate, soil and topographic conditions. To account for uncertainties in future climate projections, daily data for temperature, precipitation and solar radiation is based on five CMIP5 climate models (GFDL, HadGEM2, IPSL, MIROC and NorESM1), representing a range of temperature and precipitation changes seen in the full CMIP5 model ensemble^62^. The climate data was statistically downscaled from 30 arc-minutes to 30 arc-seconds (approximately 1 km at the equator) spatial resolution and bias corrected. Soil data is derived from the Harmonized World Soil Database (HWSD)^63^ v1.21, considering the following soil properties: soil texture, proportion of coarse fragments and gypsum, base saturation, pH content, organic carbon content, salinity and sodicity. Soil depth is considered as an additional constraint^64^. Topography data was applied from the Shuttle Radar Topography Mission (SRTM)^65^. The determinant factors are contrasted with the crop-specific requirements taken from literature^66^. The suitability is assessed by comparing the growing condition at each grid cell in terms of temperature, precipitation, solar radiation, soil properties and topography with the crop-specific requirements during the growing period. Thereby, daily climate data is used to identify an optimal growing period in the time period under consideration. Thus, the suitability approach accounts for an adaptation to changing climatic conditions. The agricultural suitability is simulated for each crop under rainfed and irrigated conditions for four different time periods, 1980–2009, 2010–2039, 2040–2069 and 2070–2099.For future time periods, we applied two climate change scenarios, RCP2.6 and RCP8.5, thereby representing the range between lower and higher emission pathways^33^. For further information on the methodology of the suitability assessment, see Zabel *et al*.^32^ and Cronin *et al*.^31^. A detailed description on the methodological improvements of the updated agricultural suitability v3.0 compared to the previous version v2.0 is provided in the description of the dataset^29^.

For our analysis, we use the maximum suitability across all considered 23 crops for each time period in order to represent a more general agricultural suitability. Besides the maximum suitability across all crops, also the agricultural suitability of each crop as well as data on the most suitable crop at each pixel are additionally provided for download^29^. We assume that current irrigation is not expanded. Therefore, we combine rainfed and irrigated suitability datasets for each time period referring to current irrigation patterns^34^.

Of the globally 78.1 million km^2^ potentially suitable for agricultural use for historic time period and under current irrigation patterns, 30% are marginally suitable (suitability values: 1–32), 59% moderately suitable (suitability values: 33–74) and 11% highly suitable (suitability values: 75–100) for crop cultivation. Since the agricultural suitability describes a general opportunity for crop cultivation and does not imply which crop is cultivated, the data does not include any assumptions on current and future (potentially shifting) production patterns. Looking at the current land-use/-cover of the suitable land according to HILDA+ land-use data^37^ for the year 2010 (Fig. 6), we see that marginally and moderately suitable land is currently mainly covered with forest and pasture/rangeland, while current croplands and urban areas are mainly located in highly suitable areas. The higher the agricultural suitability, the larger the share of cropland, which is mainly irrigated in highly suitable areas. Altogether, about half of the suitable land is under agricultural use (as cropland, pasture or rangeland) today. Urban areas are more frequently located on land highly suitable for agriculture, since in many regions humans historically preferred to cultivate fertile areas first^67^, in which they settled. Furthermore, it can be seen that the share of forests under protection decreases with higher suitability, resulting in larger areas of forests under protection on marginally suitable than on highly suitable land. Overall, the figure illustrates by how far anthropogenic dominated forms of land-use (urban areas, cropland, pasture) have already encroached on forests and natural grass- and shrublands, and it indicates that especially ecosystems on highly suitable land, mainly forests, are under higher pressure for land-use/-cover change, as a large proportion of this land has already been put under anthropogenic use.

**Table 2:** Overview of the considered data to calculate the potentially cultivable land and the potentially available cropland.

Dataset	Reference	Spatial resolution	Reference Year(s)
Suitability	Zabel, F. Global Agricultural Land Resources – A High Resolution Suitability Evaluation and Its Perspectives until 2100 under Climate Change Conditions. Zenodo 10.5281/zenodo.5982577 (2022)^29^, Zabel *et al*. 2014^32^, Cronin *et al*. 2020^31^	30 arc-seconds (~1 km)	1980–2009 2010–2039 2040–2069 2070–2099
ESA CCI land cover classification	ESA. Land Cover CCI Product User Guide Version 2. Tech. Rep. (2017). Available at: https://maps.elie.ucl.ac.be/CCI/viewer/download/ESACCI-LC-Ph2-PUGv2_2.0.pdf^38^	300 m	2010
Global Man-made Impervious Surface	Brown de Colstoun, E. C., C. Huang, P. Wang, J. C. Tilton, B. Tan. J. Phillips, S. Niemczura, P.-Y. Ling, and R. E. Wolfe. 2017. Global Man-made Impervious Surface (GMIS) Dataset from Landsat. Palisades, NY: NASA Socioeconomic Data and Applications Center (SEDAC). 10.7927/H4P55KKF. Accessed 20 05 2020^35^	30 m	2010
Protected areas	IUCN and UNEP-WCMC (2019), The World Database on Protected Areas (WDPA) [On-line], [05/2019]. Cambridge, UK: UNEP-WCMC. Available at: www.protectedplanet.net^36^	Vectors	2019
HIstoric Land Dynamics Assessment + (HILDA+)	Winkler, Karina; Fuchs, Richard; Rounsevell, Mark D A; Herold, Martin (2020): HILDA+ Global Land Use Change between 1960 and 2019. PANGAEA, 10.1594/PANGAEA.921846	0.00998837° (~1 km)	2010

### Suitability threshold

**Fig. 7:**
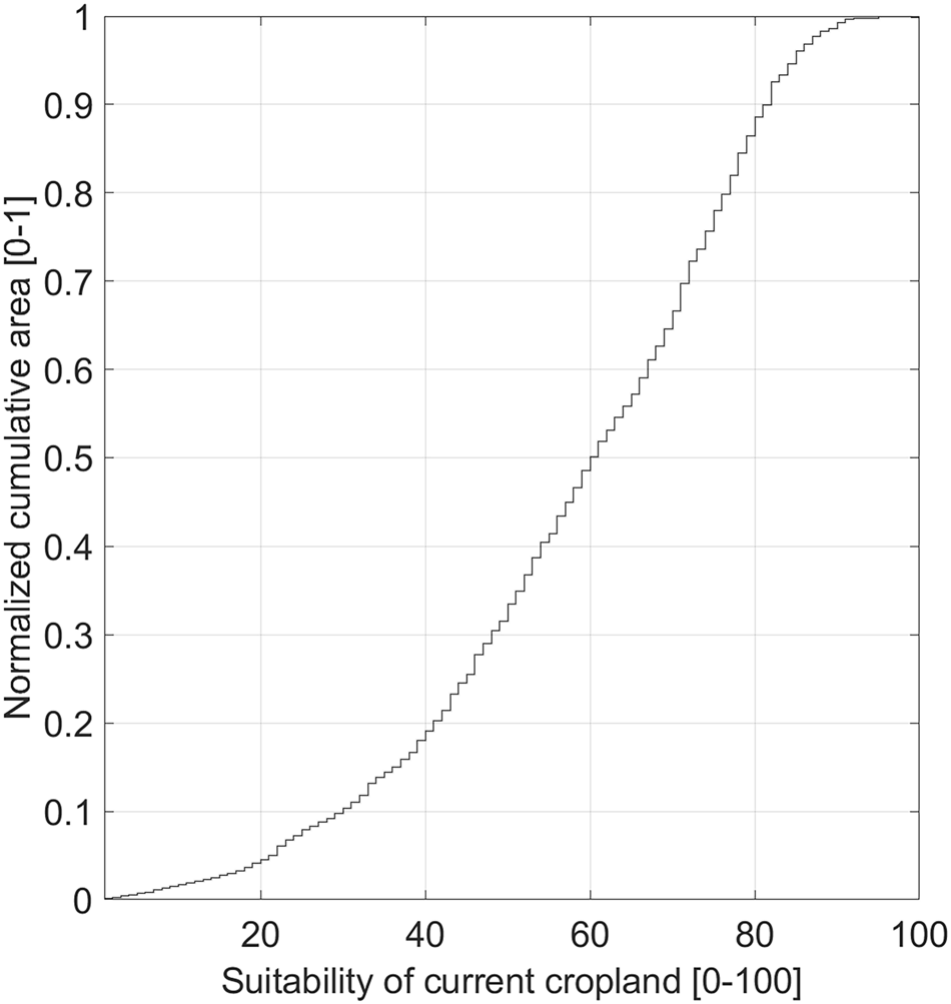
Normalized cumulative distribution function of global suitability values on current cropland area^37^. The graph displays for each agricultural suitability value the normalized cumulative current cropland area.

When referring to the agricultural suitability for our assessment of potentially cultivable land and potentially available cropland, we decided to apply no suitability threshold below which areas are excluded from our cultivable land potential. Thereby, we consider the possibility to cultivate marginally suitable land as well as the potential influence of further socio-economic factors on decisions on the agricultural use of land (see also Discussion). If a suitability threshold of e.g. 10, 20 or 30 would be applied to exclude less suitable areas, potentially suitable and potentially cultivable land would be reduced by 6%, 15% and 27% (Fig. 3) and exclude 1.6%, 4.7% and 10% of the current cropland areas (Fig. 7). Excluding land defined as marginally suitable (suitability < 33) would neglect 30% of the potentially suitable and potentially cultivable land and more than 10% of current cropland.

### Land-use and land-cover

Besides the agricultural suitability dataset, various further land-use/-cover datasets are used to calculate the potentially cultivable land and potentially available cropland (Table 2): For information on current land-cover, we refer to ESA CCI Land Cover Maps - v2.0.7^38^ for the year 2010 as baseline with 300 m spatial resolution, resampled to 1 km spatial resolution. We omit agriculturally suitable pixels that are classified as water bodies (210) or permanent snow and ice (220) to ensure consistency between the land cover and agricultural suitability. Around 598,000 km^2^ (0.76% of the global agriculturally suitable land) is thereby excluded. Moreover, wetlands, defined as shrub or herbaceous cover, flooded with fresh, saline or brackish water (180), are excluded from the potentially available cropland due to their large contribution to carbon sequestration and thus their importance for climate protection and climate change mitigation. Yet, wetlands currently already cultivated (base year 2010) are classified as cropland within the ESA CCI dataset and accordingly, for example already drained wetlands currently used as cropland are not excluded from the potentially available cropland in our approach.

To identify forest areas, we refer to the latest dataset on land-use/-cover from the HIstoric Land Dynamics Assessment + (HILDA+) by Winkler *et al*.^37^. Created by harmonizing different spatially explicit land-use/-cover information with statistical data at national scale, the forest areas for example correspond well with the Global Forest Resources Assessment (FRA) of the Food and Agriculture Organization of the United Nations (R^2^ = 0.99). This increases the applicability of our dataset in models that are calibrated with or refer to statistical forest data, such as economic models on global forest products^68^. An uncertainty assessment of the HILDA + data can be found in Winkler *et al*.^37^.

We address the often stated issue of underestimating infrastructure and settlements due to subpixel heterogeneity^20,25,27^ by using the Global Man-made Impervious Surface dataset^35^, a high spatial resolution dataset which describes the impervious cover for the year 2010 at 30 m spatial resolution. Thereby, we aim to minimize potential errors due to subpixel heterogeneity that often occur with urban areas and infrastructure^20^. For our analysis, we refer to an aggregated version of the dataset displaying the percentage of impervious cover at 1 km spatial resolution.

To consider protected areas, we refer to the IUCN and UNEP-WCMC world database on protected areas (WDPA)^36^. We exclude the most strictly protected areas, namely strict nature reserves, wilderness areas and national parks (IUCN categories Ia, Ib and II), that explicitly or implicitly do not allow for agricultural use. We assume a pixel to be not available for cropland use, if 50% or more of the pixel area is protected.

To evaluate the representation of current cropland, we refer to the HILDA + land-use/-cover dataset from Winkler *et al*.^37^. We identify 97% of current cropland as potentially cultivable land. Globally aggregated, around 1/5^th^ of the potentially cultivable land and 1/3^rd^ of the potentially available cropland is currently already under cultivation.

### Spatial structure of the result analysis

**Fig. 8:**
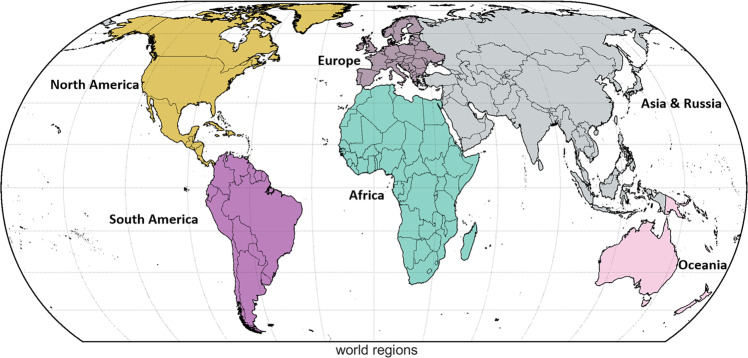
Overview of the regions referred to in the analysis of our results: Africa, Asia & Russia, Europe, North America, Oceania and South America. Regions are aggregated based on the displayed country borders according to the global administrative areas of GADM version 3.6 (https://gadm.org/).

To present and discuss our data within this study, we aggregate our results to six large world regions: Africa, Asia & Russia, Oceania, Europe, North America and South America (Fig. 8).

### Comparison to existing dataset of available cropland

Even though different datasets on potentially cultivable land are hardly comparable due to their different assumptions on availability of land for cultivation, different underlying data and differences in their spatial resolution, we can compare our datasets with the estimates of available cropland from Eitelberg *et al*.^20^, available at 5 arc-minutes spatial resolution. The ‘high estimate’ of available cropland from Eitelberg *et al*.^20^ includes croplands, mosaics of cropland and natural vegetation, open shrublands, savannas, grassland, closed shrublands and woody savannas, forests, barren or sparsely vegetated areas and also protected areas as potentially cultivable. These assumptions most closely resemble our definition of potentially cultivable land. A rather fundamental difference in the datasets is the general assumption of Eitelberg *et al*.^20^ that on average 15% of a raster cell is occupied by nonproductive uses.

Comparing our potentially cultivable land for historic time period with the ‘high estimate’ from Eitelberg *et al*.^20^, we see that our potentially cultivable area is with 77.7 million km^2^ around +48% larger than the ‘high estimate’ of 53.3 million km^2^ potentially cultivable land. More than half of this additionally included land (53%; around 12 million km^2^) can be considered as only marginally suitable for agriculture. Yet, 42.5% is moderately suitable and 4.5% are even highly suitable areas, altogether leaving around 10 million km^2^ of land relatively well suitable for cultivation excluded in the estimate by Eitelberg *et al*.^20^ The largest additional area, around 7 million km^2^, can be found in Africa, where our approach additionally includes mainly marginally suitable land in the Sahel and the east and south of Africa, such as parts of Niger, Sudan, Somalia Angola, Namibia or Botswana. However, around 45% of the additionally included potentially cultivable land is moderately or highly suitable, for example areas in the Democratic Republic of the Congo, Ethiopia or Zambia. In Oceania, our potentially cultivable land is more than twice as large as the estimate from Eitelberg *et al*.^20^: 3.2 million km^2^ are additionally included, of which around 48% are moderately or highly suitable. Thereof, 80% is located in Australia, where our approach identifies around 2.8 million km^2^ of additionally potentially cultivable land, of which 45% are moderately or highly suitable. In Asia & Russia, we additionally include 5.3 million km^2^, of which 8% are highly and 46% moderately suitable for agriculture. Applying a suitability threshold to our potentially cultivable land would reduce its extent and might bring it closer to the estimate from Eitelberg *et al*.^20^, but would on the other hand also exclude areas which are currently already used as cropland.

## Supplementary information

Supplementary Information

## Data Availability

The datasets on potentially cultivable land and potentially available cropland are available under: Schneider, J.M., Zabel, F., Mauser, W. Global inventory of potentially cultivable land and potentially available cropland under different scenarios and policies. *Zenodo* 10.5281/zenodo.5993934 (2022)^30^. The potentially cultivable land and the potentially available cropland are both available for historic (1980–2009) and future (2010–2039, 2040–2069 and 2070–2099) time period under RCP2.6 and RCP8.5, and under current irrigation patterns as well as for rainfed and irrigated conditions separately. Thus, different assumptions on the expansion of irrigation patterns could be applied by users. All datasets are available referring to the agricultural suitability of all considered 23 crops, as well as referring only to the agricultural suitability of the 17 food, feed, fiber and first-generation bioenergy crops, thereby excluding land that is solely suitable for second-generation bioenergy crops (see Table 1). The datasets provide the potentially cultivable land and the potentially available cropland in km^2^ at 30 arc-seconds and 30 arc-minutes spatial resolution and aggregated to country level according to the global administrative areas of GADM version 3.6.

**Table:** 

**Time period**		**Climate change**	**Irrigation**	**Considered crops**
*historic*	1980–2009	—	■ Rainfed ■ Irrigated ■ Current irrigation patterns	■ All 23 crops ■ 17 crops excluding second-generation bioenergy crops
*future*	2010–2039	RCP 2.6	■ Rainfed ■ Irrigated ■ Current irrigation patterns	■ All 23 crops ■ 17 crops excluding second-generation bioenergy crops
		RCP 8.5		
	2040–2069	RCP 2.6	■ Rainfed ■ Irrigated ■ Current irrigation patterns	■ All 23 crops ■ 17 crops excluding second-generation bioenergy crops
		RCP 8.5		
	2070–2099	RCP 2.6	■ Rainfed ■ Irrigated ■ Current irrigation patterns	■ All 23 crops ■ 17 crops excluding second-generation bioenergy crops
		RCP 8.5 ■ Rainfed ■ Irrigated ■ Current irrigation patterns ■ All 23 crops ■ 17 crops excluding second-generation bioenergy crops ■ Rainfed ■ Irrigated ■ Current irrigation patterns ■ All 23 crops ■ 17 crops excluding second-generation bioenergy crops ■ Rainfed ■ Irrigated ■ Current irrigation patterns ■ All 23 crops ■ 17 crops excluding second-generation bioenergy crops ■ Rainfed ■ Irrigated ■ Current irrigation patterns ■ All 23 crops ■ 17 crops excluding second-generation bioenergy crops The suitability data (version 3.0) is available for all crops, climate change scenarios, irrigation assumptions and time periods under: Zabel, F. Global Agricultural Land Resources – A High Resolution Suitability Evaluation and Its Perspectives until 2100 under Climate Change Conditions. *Zenodo* 10.5281/zenodo.5982577 (2022)^29^.

## Code availability

The MATLAB code for calculation and creation of the dataset is available as supplementary information file.
