# Supplementary material for: Global inventory of suitable, cultivable and available cropland under different scenarios and policies
Source: Sci Data. 2022 Aug 27;9:527. doi: 10.1038/s41597-022-01632-8 (PMC9420104; doi:10.1038/s41597-022-01632-8)
Supplement: Supplementary file 1 — Supplementary Information [file 41597_2022_1632_MOESM1_ESM.pdf]

```
%% Calculation of potentially cultivable land and potentially available cropland
% by Julia M. Schneider, 2022
% Contact information: Schneider.ju@lmu.de, Department of Geography, Ludwig-Maximilians-
Universität München (LMU), Munich, Germany.

%% ----- calculate potentially cultivable land and potentially available cropland -----%%
% for all 23 crops or for a subset excluding second-generation bioenergy crops (Jatropha,
Miscanthus, Switchgrass, Reed canary grass, Eucalyptus and Willow)
% under current irrigation patterns and for rainfed and irrigated conditions separately

% potentially cultivable land (PCL)
% = area that is suitable for agriculture (Zabel et al. 2022) excluding open water
bodies, permanent snow and ice and man-made impervious surfaces

% potentially available cropland (PAC)
% = potentially cultivable land excluding strictly protected areas, forests and wetlands

suitability_th=0; % define suitability threshold above which pixels are suitable for
agriculture

%% Exemplary calculation for historic time period (1980-2009)
rcp=char('hist');
climtimeframe=char('1980-2009');
for crops={'allcrops','1to17'} % define whether the suitability of all crops is taken
into account (allcrops) or second-generation bioenergy crops are excluded (1to17)
  if strcmpi(crops,'allcrops') % define according filenames of suitability
    suitcrops={'1'};
  else
    suitcrops={'1to17'};
  end

  for irrigation={'rainfed','irrigated','irrigationpatterns'}
    for scenario={'PCL','PAC'}
      scenario=char(scenario);
      if strcmpi(scenario,'PCL')==1 % potentially cultivable land: no restrictions into
      forests, wetlands or protected areas
        forest=[];
        wetlands=[];
        protected_areas=[];
      elseif strcmpi(scenario,'PAC')==1 %  potentially available cropland: protected
      areas, forests and wetlands are excluded
        forest=[44]; % HILDA + land cover class forest
        wetlands=[180]; %ESACCI land cover class wetlands
        protected_areas=[3,4,5]; % Code of IUCN Categories that are excluded (Ia (strict
        nature reserve, Value:4), Ib(wilderness area, Value:5) and II(national park,
        Value:3));
      end



      %% Load Data
      pixelarea1km=load('pixelarea_1km.mat').pixel_area_km; % pixel area 1 km (in km2)

      % suitability for specific timeframe (historical, RCP2.6, RCP8.5) for irrigated,
      rainfed, or with current irrigation for all crops (1) or 1 to 17

      suitability=readbil(['suitability\',char(irrigation),filesep,climtimeframe,'_',rcp,'
      \overall_suitability_1\overall_suitability_',char(suitcrops),'.bil']);
      suitability(suitability==255)=0; %255=no data

      % ESACCI land-use/-cover 1 km (2010)
      ESACCI=readbil('ESACCI2010.bil');
```

```
      water=[210,220]; %  land use classes water bodies(210), permanent snow and ice(220)

      % HILDA + land-use/-cover 1 km (2010)
      HILDAplus=geotiffread(['hilda_plus_2010_states_GLOB-v1-0_wgs84-nn.tif']);

      % GMIS: % of impervious surface 1km (2010)
      [GMIS, GMIS_metadata]=geotiffread('GMISraster.tif');

      % WDPA protected areas 1km (2015)
      [WDPA, WDPA_metadata]=geotiffread('WDPA.tif');


      %% Calculate available cropland area 1km
      % loop over 1 km raster
      avail_crop=double(zeros(size(suitability,1),size(suitability,2)));
      for row=1:size(suitability,1)
        disp(['Row ',num2str(row),' out of ', num2str(size(suitability,1))]);
        for col=1:size(suitability,2)
            if suitability(row,col)> suitability_th &&
            ismember(WDPA(row,col),protected_areas)==0 &&
            ismember(ESACCI(row,col),water)==0 && ismember(HILDAplus(row,col),forest)==0
            && ismember(ESACCI(row,col),wetlands)==0% suitable and no snow, ice or water,
            not protected or covered with forest or wetland
              avail_crop(row,col)=pixelarea1km(row)*((100-GMIS(row,col))/100);
            else % if not suitable or covered with snow, ice or water (or covered with
            forest or classified as protected area): no potentially cultivable land/
            available cropland
              avail_crop(row,col)=0;
          end
        end
      end
    end
  end
end
```
